# The Application of Microencapsulation Techniques in the Treatment of Endodontic and Periodontal Disease

**DOI:** 10.3390/pharmaceutics3030538

**Published:** 2011-08-26

**Authors:** Asteria Luzardo Álvarez, Francisco Otero Espinar, José Blanco Méndez

**Affiliations:** 1 Departamento de Farmacia y Tecnología Farmacéutica, Facultad de Ciencias, Universidad de Santiago de Compostela, 27002 Lugo, Spain; E-Mail: jose.blanco.mendez@usc.es; 2 Departamento de Farmacia y Tecnología Farmacéutica, Facultad de Farmacia, Universidad de Santiago de Compostela, 15782 Santiago de Compostela, Spain; E-Mail: francisco.otero@usc.es

**Keywords:** microencapsulation, periodontal diseases, endodontic failure, periodontal pocket, root canal, controlled release

## Abstract

In the treatment of intracanal and periodontal infections, the local application of antibiotics and other therapeutic agents in the root canal or in periodontal pockets may be a promising approach to achieve sustained drug release, high antimicrobial activity and low systemic side effects. Microparticles made from biodegradable polymers have been reported to be an effective means of delivering antibacterial drugs in endodontic and periodontal therapy. The aim of this review article is to assess recent therapeutic strategies in which biocompatible microparticles are used for effective management of periodontal and endodontic diseases. *In vitro* and *in vivo* studies that have investigated the biocompatibility or efficacy of certain microparticle formulations and devices are presented. Future directions in the application of microencapsulation techniques in endodontic and periodontal therapies are discussed.

## 1. Introduction

Endodontic and periodontal pathologies are closely related to each other and as primary diseases each may have secondary effects on the other. The appropriate treatment and route of administration are selected according to the differential diagnosis and etiopathogenesis of each disease. Periodontal and endodontic therapies can be grouped into three broad categories: mechanical debridement and cleaning to eliminate bacteria; treatment aimed at killing or affecting the metabolism of the infectious organism, such as antiseptics and antibiotics, and treatment that affects the environment of the infectious organism [1,2]. Antiseptic or antibiotic drug-loaded devices can be used to treat infection, or as a pharmacological complement to mechanical treatment by introducing particulate formulations into the targeted sites. New approaches involve the use of local drug delivery systems based on microparticles made from biocompatible polymers. Such devices enable the introduction of antimicrobial agents or other drugs directly in the periodontal pocket, or inside the root canal, and the prolonged release of constant concentrations of these agents for better control of infections. Complementary use of drug-loaded microparticle formulations can reduce periodontal and endodontic failures, and even partly replace surgical or mechanical treatment, especially in refractory patients.

Biodegradable microparticles have been widely studied as drug delivery systems for many bioactive compounds such as low molecular weight drugs, macromolecular substance, DNA and antigens [3-5]. Synthetic or natural polymers have been considered for preparation of microspheres for administration by the nasal, pulmonary, oral or parental route [6-8]. Because of their excellent biocompatibility characteristics, natural polymers such as chitosan and other polysaccharides, collagen, hyaluronic acid and cellulose are often used for microsphere preparation. Poly(*D*,*L*-lactide-co-glycolide) (PLGA) biodegradable polymers also have a long history as drug delivery systems in many biomedical fields. Their biodegradability, excellent biocompatibility and ability to provide controlled-drug release have led to them being used in microsphere preparation [9-11]. Other examples of biodegradable polymers used in microparticle preparations include polyanhydrides, poly-ε-caprolactone and polyphosphazenes [12-14]. Biodegradable polymers, of natural or synthetic origin, can be degraded into acceptable biocompatible products by chemical or enzymatic hydrolysis. Microencapsulation of therapeutic agents has traditionally been performed by three main techniques and their modifications: spray-drying, solvent extraction-evaporation and coacervation or phase separation. These and other methods are described by [9,11,15-20]. Other reviews dealing with diverse aspects of microspheres for drug delivery are available [21-23].

Localized administration of medication by use of polymeric microspheres offers the advantage of providing rate-controlled release of drug at the site of action, maintaining the concentration in the therapeutic window (thus avoiding systemic side-effects), and protection of the substance of interest from degradation and physiological clearance. The main factors responsible for controlling the rate of drug release from microparticles include physicochemical characteristics such as size, chemical composition, morphology and the rate of degradation of polymers and drug. The full therapeutic potential of microparticles in pharmaceutical applications lies in their particular properties: large surface area, ability for site-controlled drug delivery, the wide variety of biocompatible polymers that can be used, simplicity of implantation at the site of delivery, and charge density. Moreover, all these parameters can be modified to design customized microparticulate systems adapted to the requirements of each clinical case.

This review focuses on localized controlled-release polymer microspheres used in periodontic and dental applications. We begin with a general description of periodontal diseases (PD) and endodontic failure (EF) and the conventional treatments used. We present the most recent therapeutic strategies based on microencapsulated drugs, with the main emphasis on periodontal and dental routes of administration. The properties of drug-loaded polymeric microparticles enable prolonged delivery of large amounts of drugs, mainly antibiotics, through the periodontal and dental route of administration, thus reducing the side effects of systemically applied antimicrobials in the treatment of PD and EF. The current status of studies and recently reported approaches evaluating microparticulate formulations for endodontic and periodontal applications, advantages over other systems for infection inhibition, practical difficulties, localized delivery for periodontal and dental tissue regeneration and safety issues are further aspects that will be discussed.

### 1.1. Periodontal Diseases (PD) and Periodontal Treatment

**Figure 1.**
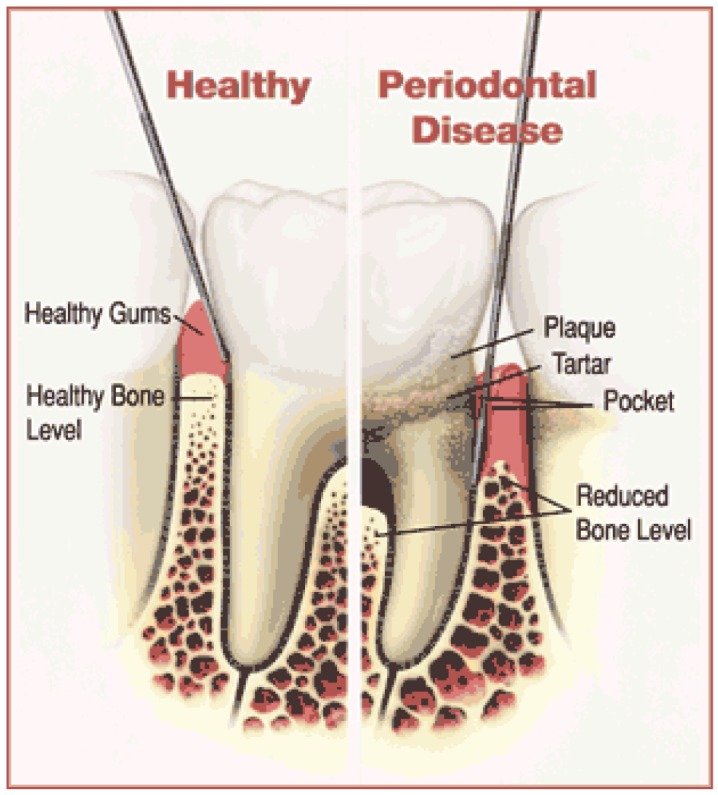
Representation of the structure of a healthy tooth and how it is typically affected by periodontal disease (picture source: American Academy of Periodontology; http://www.perio.org).

Periodontal disease is a wide term that covers a combination of multiple inflammatory diseases affecting the tissues surrounding teeth: gum tissue, alveolar bone and the periodontal ligament, which is composed of collagen fibers (Figure 1). The prevalence of peridontitis worldwide has been determined on the basis of differences in clinical diagnosis between different forms of periodontitis (gingivitis, chronic or aggressive periodontitis), although according to the epidemiological data, the occurrence of this PD is related to oral hygiene status and socio-economic class. In the USA, the prevalence of chronic periodontitis in adults is around 35% [24], whereas in Western Europe 13–54% of the population is affected by periodontal diseases [25]. In low income countries, the percentage of the population affected is around 45%. Several factors affect clinical signs of PD, such as tobacco use, general health, pregnancy, diabetes and social conditions [26-28].

In periodontal pathologies, the supporting structures become infected and necrotic, leading to loss of attachment of teeth. Periodontal pathology is an infectious process with different degrees of severity: gingivitis, mild periodontitis, moderate periodontitis and advanced periodontitis, with extensive periodontal breakdown. Clinical signs of periodontitis include gum swelling, bleeding, pus discharge, tooth mobility by changes in gingival tissues and formation of a periodontal pocket. Periodontitis is essentially irreversible and if left untreated, moderate periodontitis can lead to the loss of tooth by the degradation of collagen by bacterial enzymes. The periodontal pocket where the tooth connective tissue attachment has been gradually destroyed provides an excellent environment for pathogenic bacteria. The periodontal microbiota adheres to the tooth inducing inflammation of the surrounding soft tissues. The inflammatory and immune mediators produced by the host in response to bacterial activity also enhance destruction of the periodontal tissue [29-31]. Periodontal diseases involve the formation of a biofilm on the tooth surface. Dental biofilm is also known as “plaque”. In this context, a biofilm is broadly defined as sessile and mixed-bacterial cells that adhere to each other and to a surface forming enclosed matrices composed of polymers of their production [32-35]. Growth of microbial biofilms is always associated with resistance to antimicrobial agents [36]. Thus, the plaque biofilm is considered the main factor that causes several localized diseases in the oral cavity, including periodontal disease, dental caries and endodontic failure. The aim of periodontal procedures is mainly to eliminate bacterial deposits or dental plaque from the tooth surface by mechanical treatment in combination with good oral hygiene to prevent re-infection of the subgingival area by pathogens and consequently, to preserve the tooth. Elimination of periodontopathic microorganisms by systematically administered antimicrobial agents has been widely used as adjunctive therapy to mechanical procedures used to treat periodontitis such as scaling and root planning (SRP).

However, antibiotics applied locally in the periodontal pocket may further eliminate pathogens and thus enhance the effect of conventional surgical therapy without the side effects of systemically administered antibiotics. Local administration of antibiotics necessarily involves insertion of the drug-loaded device in the periodontal pocket, to achieve effective concentrations at the target site. The enhancement of antibiotic penetration into biofilms is of particular importance to prevent microbial colonization in periodontal pockets and dental root canals, since the drug should reach sites of difficult access. The use of sustained drug delivery systems may also provide drug delivery over a prolonged period in periodontal pockets. In this context, polymeric microparticles may be of particular interest by virtue of their ability to achieve sustained delivery of effective biocidal levels of drug, to inhibit microbial adhesion and to be retained or adhered on the dentin or biofilm surface, as well as their biocompatibility and large surface area to volume ratio. In addition, the small size of microspheres enables them to penetrate into deeper lesions in severe periodontitis. It is also possible to modify the physicochemical characteristics of microparticles, such as surface charge, size, porosity, hydrophobicity and even introduce ligands with functional groups on the surface [37,38]. Drug-microparticles in periodontal pockets may well provide useful antimicrobial activity to control or inhibit infection in both hard and soft tissues.

### 1.2. Dental Disease and Endodontic Re-Treatment

Partial or total tooth loss through apical periodontitis caused by acidogenic bacteria is a major health problem. Endodontic disease is a common chronic disease that affects all age groups, has a significant impact on quality of life and is costly to treat [39] accounting for between 5–10% of health care expenditure [25,39]. Epidemiological surveys of dental health conditions in different countries have shown that the level of severity of the prevalence of caries is about 50% in well-developed countries, whereas in low income countries, the prevalence is around 90% [40]. Periodontal infections are the most frequent type of periodontal diseases, and are responsible for 75% of adult tooth loss.

**Figure 2.**
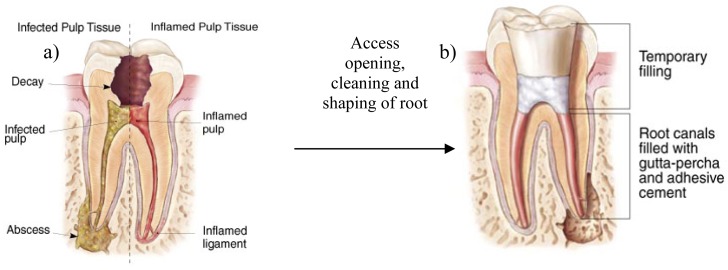
Inflammation and infection of pulp tissue (**a**) and example of completed root canal treatment. The temporary filling is placed into the empty pulp chamber; (**b**) Adapted from http://www.aae.org/Patients/Endodontic_Treatments/Root_Canals.aspx

Endodontics or root canal therapy is the part of dental science that is indicated for patients who have already undergone endodontic therapy, but who suffer from persistent infections. It is of particular concern that resistant microbial species such as *Enterococcus faecalis* (the most common) [41,42], *Staphylococcus aureus*, *Pseudomonas aeruginosa, Bacillus subtilis*, Streptococcus *spp., Actinomyces spp.* and *Candida spp.*, among others may remain in root canals [43-45]. Bacteria can colonize the enamel of the tooth surface, as well as the supporting structures of teeth. Several microbial species can attach to the tooth surface and form a dense polymeric structure involved in surface adhesion and host invasion [46-50]. Endodontic biofilms represent a common cause of persistent infections in the root canal because of their resistance to antimicrobial compounds [47]. If not detected early, caries leads to erosion of the enamel and the tooth pulp. The tooth pulp forms the dentin and contains the nervous and vascular system that supplies nutrients to the tooth. The pulp also includes cells such as fibroblasts, odontoblasts, mast cells, macrophages and plasma cells. Over time, the irreversible infection affects the pulp tissue and the tooth become non-vital as a result of necrosis. Management of the injured tooth to preserve it as a healthy and functional unit (so-called root canal treatment) includes removal of the damaged tissue, and cleaning and drying the root canal cavity. This step does not involve the use of intracanal medication. The root canal is then dressed with irrigant antimicrobial solutions (sodium hypochlorite, chlorhexidine, iodine-based irrigants, EDTA) and a provisional cap is inserted to separate the root canal from the oral cavity. This procedure is repeated every 7–10 days until the treatment is completed. The final step consists of sealing the root canal with a permanent cap to prevent further contamination by microorganisms (Figure 2). The success of endodontic treatment basically depends on the effective elimination of inflamed and/or infected pulp tissue from the root canal system. Most of the infecting bacteria may be reduced by the endodontic procedure.

Root canal treatment involves mechanical and chemical activity to reduce the bacterial load in the root canal system. Although most infecting pathogens are removed during the chemo-mechanical procedure, residual bacteria can survive within the dentinal tubules [51,52]. In endodontics, the development of a bacterial biofilm within the root canal system is also a key cause of endodontic failure, whereby necrosis in the surrounding tissues leads to endodontic failure.

The endodontic treatment therefore fails due to resistance to antibiotics, unsuccessful sealing of the canal or formation of a bacterial biofilm within the root canal cavity [31]. The complex anatomy of the root canal system, with accessory and lateral canals that are part of the root canal system and poor tissue penetration of the systemic antibiotic therapy are important factors in the complete debridement of bacteria and subsequently, in endodontic failure.

Although the antimicrobial efficacy of conventional irrigants is generally recognized, they may produce some complications such as tissue irritation or hypersensitivity. Sodium hypochlorite, normally used at concentrations of 0.5–5% [53,54] can produce pain, swelling, haemorrhage and even secondary infections as a result of accidental injection [55-57], or perforations due to the use of large volume of irrigants extruded from the canal. The ability of calcium hydroxide to eliminate microorganisms in the root canal is not reliable, especially for some bacteria in particular [58-60], and inflammatory reactions have been reported [61]. Chlorhexidine has shown an important degree of antimicrobial activity in comparison with other intracanal medication [62]. However, long-term sustained release of the drug is necessary for it to be effective [63,64], and it may be affected by other anionic components of the formulation, rendering it inactive. The use of irrigants with antimicrobial properties provides a local drug effect but this cannot be sustained to maintain therapeutic levels of drug over several days.

The difficulty in eliminating *Enterococcus faecalis* and other bacteria that infect dental root canals has led to interest in the development of alternative formulations capable of sustained release of antibiotic within the canal, between the preparation and obturation phase, as inter-appointment medication.

## 2. Microparticulate Delivery Systems and Periodontal Diseases

Antimicrobial or antiseptic agents are applied locally for the treatment of periodontitis in combination with mechanical root debridement. Subgingival irrigation with drug solutions provides high concentrations in the periodontal pocket for only short periods. In consequence, repeated oral irrigation is required to exert bactericidal or bacteriostatic effects. For use of the periodontal route as a site for administration of drug-loaded microparticles, the amount of drug absorbed across a membrane is directly proportional to the surface area of exposure, the applied drug concentration, the permeability coefficient of the drug and the residence time. In a pathological situation, the turnover rate of the medium volume of a moderately-sized pocket (0.5 μL) is around 40 times per hour [65]. If we compare this with the salivary flow inside the oral cavity, 28 times an hour, the rapid clearance of substances from the periodontal pocket is obvious. Goodson [65] also suggested that this rapid turnover was related to the short duration of action of irrigation treatments. In fact, the high rate of clearance represents the main hurdle to maintaining effective concentrations of drug within the periodontal pocket. The use of microparticulate systems for controlled drug release may well improve the antibiotic efficacy in the periodontal pocket, with consequent clinical benefits. Periodontal pockets may be used as natural sites for easy placement of microparticulate delivery systems. Bearing in mind that the average depth of a periodontal pocket is about 5–8 mm, in practice, the maximum size of a periodontal delivery system should not be any larger than this. The physical space in the periodontal pocket therefore represents another limitation to the release of the active substance to counteract the continuous loss due to gingival crevicular fluid flow. The gingival crevicular fluid provides the release medium for the drug from microspheres throughout the periodontal pocket. As an intra-pocket device for antibiotic drug administration microparticles represent an attractive way of maintaining the effective drug concentration in the gingival crevicular fluid in a sustained manner for prolonged periods, in order to obtain therapeutic benefits. A microparticulate delivery system for periodontal healing should ideally display high biocompatibility, provide controlled release of the active substance, be easy to implant (e.g., with a syringe), provide drug stability and should be retained in the periodontal pocket.

The periodontal pocket has been investigated as a site for local drug delivery by many research groups, and the route has already achieved commercial status with several drugs such as clorhexidine gluconate (Periochip^®^, Dexcel Pharma) and tetracycline (Actisite^®^, P&G/Alza). Extensive research has been directed towards the development of intrapocket devices including films [66,67], sponges [68], strips [69-71], fibers [72,73], gels [74,75], semisolid form [76], chips [77,78], nanoparticles [79-81] and microparticles [77,82]. A full review of other kind of drug delivery systems apart from microparticles is beyond the scope of this article and it is extensively covered in several reviews [83-85].

Multiparticulate systems used in the periodontal delivery of drugs mainly include microparticles and nanoparticles. In addition to the inbuilt advantages to multiparticulate drug delivery systems (*i.e.*, safety, uniformity, reproducibility), these systems have the advantage that they can be incorporated in usual oral formulations as suspensions or toothpastes, or in new drug delivery systems such as bioadhesives or sensitive hydrogels, or can even be directly injected into the periodontal cavity to achieve effective control of drug release. In general, microparticles have been found to be a good approach for local treatment of periodontal infection, and the implementation of bioadhesive properties may increase their therapeutic potential to avoid leaching from the periodontal pocket.

One of the first studies describing the use of microspheres in the periodontal cavity was developed by Folke and Stallard in 1967 [86]. Radiolabeled polystyrene tracer microspheres (15 μm) were injected into the right external carotid artery of monkeys, with the aim of studying periodontal microcirculation. However, it was during the 1990s that different formulations based on microparticles were developed for drug delivery in periodontal treatments, mainly for controlled delivery of antibiotics, antiseptics, antinflammatory agents or growth factors. Since non-biodegradable materials require complete removal of the device at the end of the treatment, most research efforts have focused on the development of microparticles from biodegradable materials. Different materials and preparation methods have been used to prepare biodegradable microparticles for periodontal delivery. In fact, a wide variety of biodegradable materials are available for microencapsulation, including polyester polymers (*i.e.*, poly *L*-lactide, poly *D*,*L*-lactide, polyglycolide and copolymers, polycaprolactones), polyanhydrides (*i.e.*, poly adipic anhydride, carboxyphenoxypropane copolymerized with sebacic acid, poly-methyl vinyl ether-co-maleic anhydride), polyamides, polyalkylcyanoacrylates, or natural macromolecules such as proteins (*i.e.*, albumin, globulin, gelatin, collagen, casein) or polysaccharides (starch, cellulose, chitosan, dextran, alginic acid) [87,88].

One of the synthetic materials most commonly used in the preparation of biodegradable microparticles for periodontal delivery is the polymeric ester polylactide (PLA) and polylactide-co-glycolide (PLGA). The advantages of these biodegradable poly(α-hydroxyacids) are that they display low toxicity, and undergo slow biodegradation, which determines the release kinetics of the polymer. Drug release can be modulated by modifying the polymer ratio (lactide:glycolide), molecular weight and crystallinity. PLA and PLGA microparticles have been used for periodontal release of tetracycline [82,88], minocycline [89-91], chlorexidine [92], doxycycline [93,94], ipriflavone, histatins, insulin-like growth factor-1 [95], alendronate [96], platelet-derived growth factor-BB (PDGF-BB) [95] and triclosan [79]. Other synthetic materials assayed for periodontal delivery include poly-ε-caprolactone (PCL) used for encapsulation of doxiciclin [93] and fabrication of scaffolds via PCL microsphere agglomeration [97], and polyphosphazenes for microencapsulation of naproxen and succinylsulphatiazole [98].

Natural polymers display low or no toxicity, low immunogenicity, and thereafter good biocompatibility. As a result, natural polymers are often preferred to synthetic polymers because although the latter have adequate properties (stability, reproducibility, low toxicity), the former show better biocompatibility [99,100]. Naturally occurring biocompatible polysaccharide are often used in the preparation of biodegradable microparticles for periodontal delivery. Chitosan is a polysaccharides widely used in the elaboration of periodontal microparticles as a main component or in mixtures with other materials such as alginates. This cationic polysaccharide has bioadhesive properties and can bind to negatively charged mucosal cell surfaces. Chitosan has been used to encapsulate minocycline [11,101], tetracycline [68,102,103], triclosan [104], alendronate [96], antisense oligonucleotide [81] and human periodontal ligament fibroblasts [105]. Alginate is another natural polysaccharide used extensively to treat periodontitis. This material has been used in combination with hydroxyapatite to prepare microspheres containing amoxicillin, amoxicillin-clavulanic acid or erythromycin [106]. In Table 1, we provide a few examples of polymeric materials that were been used for periodontal applications [82,91,93,99,101,102,107-110,116-118,123,125,126].

In 1988, Baker *et al.* [111] proposed the use of PLGA microspheres loaded with tetracycline suspended in polyethylene oxide/polypropylene oxide tri block copolymers (PEO-PPO-PEO Poloxamer 407 or Pluronic F127) for treatment of periodontal infections. PEO-PPO-PEO are amphiphilic polymers capable of self-aggregation in solution and of forming reversible thermo-sensitive hydrogels. Poloxamer 407 aqueous solutions are syringable liquids at ambient temperature, but turn into a viscoelastic gel once administered in the periodontal pocket, as a result of the increased temperature. The gelation process increased the time of permanence of the formulation at the application site. This system was found to be able to maintain tetracycline concentrations above therapeutic levels for about 4 days in *in vivo* studies in monkeys.

In 1997, Jeyanthi *et al.* [112] proposed the microencapsulation of the antimicrobial peptide histatin P-113, for the treatment of periodontitis. Controlled-release-microspheres were prepared using a low molecular weight uncapped glycolide-lactide copolymer. Release studies showed loss of the activity of the peptide released from the PLGA microspheres. Nevertheless, addition of the nonionic surfactant Poloxamer 407 during elaboration of the microspheres retained the peptide activity in the system, even after the drug was released.

The antibiotic tetracycline-HCl has often been used in microparticle systems as it has shown good clinical efficacy in periodontal infections. However, although numerous tetracycline-loaded microparticle formulations have been developed and characterized in vitro, very few studies have aimed to determine the clinical efficacy in appropriate animal models. For example in 1999, Sendil *et al.* [108] used a biodegradable microbial polyester, poly(3-hydroxybutyrate-co-3-hydroxyvalerate) (PHBV) with modified valerate content to design microspheres and microcapsules loaded with tetracycline. Tetracycline was encapsulated in the PHBV microspheres and microcapsules both in its acidic and in neutral form by a w/o/w double emulsion method, to investigate the effect of the concentration of two emulsifiers, polyvinyl alcohol (PVA) and gelatin, on the encapsulation efficiency, loading, release characteristics and morphological properties. Drug release was obtained by diffusion mechanisms but no effect on biodegradation rate was observed in the release. However, so far, no *in vivo* studies have been performed to evaluate the potential clinical applications.

**Figure 3.**
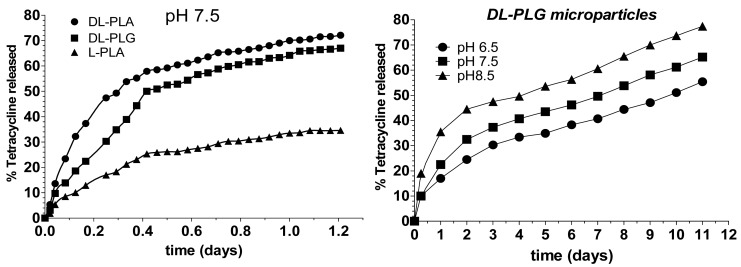
Tetracycline release profiles obtained from microparticles elaborated with DL and L- PLA and DL-PLG using a release buffer of pH 7.5 (right) and from DL-PLG microparticles in tree buffers with different pH. Modified with permission from Informa Healthcare [82].

In 1997, Esposito *et al.* [82] also prepared biodegradable tetracycline loaded-microparticles with poly-ortoester polymers, for periodontal disease therapy. In this study, microparticles were made with L-PLA, DL-PLA and DL-PLGA by different methods. The emulsion evaporation technique was used as the preparation method, and a concentrated salt solution was used as external phase to increase the loading efficacy of tetracycline. The incorporation of gelatin as a stabilizer provided the best results in terms of morphology and encapsulation efficiency in both cases, for drug base and salt. *In vitro* experiments demonstrated the ability of the microparticles to release the tetracycline content slowly over two weeks, and the release kinetics were mainly influenced by the type of polymer (Figure 3). As previously reported, no *in vivo* studies have been developed to test the efficacy of the formulation for periodontal treatment.

Other strategies have been used to produce bioadhesive periodontal systems. For example, Govender *et al.* [102,103] proposed the preparation of biodegradable microspheres by ionotropic gelation of chitosan loaded with tetracycline. To optimize features of microparticles such as bioadhesiveness and drug release, a method of experimental design optimization (Box-Behnken experimental design) was used. Formulation parameters were also optimized to enhance the tetracycline loading. Drug release and antimicrobial studies revealed that microparticles released the minimum concentration required to inhibit growth of microorganisms over a period of 8 h, although this was still not reliably effective in *in vivo* studies.

Minocycline is one of the antibiotics most frequently used for treatment of periodontal diseases. At present a commercial formulation based on minocycline loaded-microspheres is available on the market under the registered trade name of Arestin^®^ (Orapharma Inc.). One of the first uses of minocycline microcapsules was described by Lawter *et al.* [90], who in 1990 administrated minocycline microcapsules as a powder in the periodontal pocket of beagle dogs and obtained an effective concentration of antibiotic for at least one week. In 1995, Park *et al.* [113,114] developed chitosan-encapsulated alginate microspheres loaded with 10% minocycline as a biodegradable device that could be implantable in the periodontal pocket. Microcapsules were prepared by ionotropic gelation and designed to maintain drug concentrations for a week in the gingival fluid. Microcapsules showed zero order release kinetics with an initial burst effect. Microcapsules were injected in 15 patients as an adjunct to supragingival scaling and compared with scaling and root planing treatment alone. Microbiological indicators of periodontal health were better at minocycline sites than when scaling and root planing was used alone [114]. A similar system was proposed in 1996 by Lee *et al.* [115], who prepared minocycline-HCl loaded-microcapsules made from alginate and chitosan, capable of releasing the antibiotic for 7 days above its MIC_90_.

In clinical studies in 2001, Williams *et al.* [91] performed a controlled trial to evaluate the efficacy of the local administration of minocycline loaded-PLGA microspheres in the treatment of periodontal disease. A multicentre study, including nearly 750 subjects with moderate or advanced periodontitis was carried out in 18 centres, with primary outcome measured as the reduction in probing depth after 9 months. Clinical results demonstrated the superiority of locally delivered minocycline PLGA microspheres over nine months over SRP alone or SRP plus vehicle. In addition, the local administration of minocycline PLGA microspheres led to a potentially short term reduction in the levels of the gingival crevicular fluid biomarker interleukin 1-beta (IL-1), a potent bone-resorptive cytokine associated with periodontal diseases activity [116].

Studies for determining the clinical efficacy of microspheres containing minocycline (Arestin) in comparison with that of a chlorhexidine gel as an adjunct to mechanical debridement of incipient peri-implant infections was carried out by Renvert *et al.* [117,118]. The results demonstrated that local use of the microencapsulated antibiotic as an adjunct to mechanical treatment was effective for peri-implant lesions, with better results than with chlorhexidine gel. Clinical benefits were also obtained by the use of minocycline microspheres with a sustained release over 12 months. In contrast, no advantages were obtained with a combined mechanical/minocycline treatment in comparison with the minocycline microsphere treatment. However, the authors indicated that treatment may have to be repeated. Finally, the efficacy of minocycline microspheres on the treatment of different phases of the periodontal disease as adjuvant to scaling and root planing [116,119-122] or surgical therapy have been demonstrated in other clinical studies [109,123]. Also, the local administration of drug-loaded microspheres has been shown to reduce the bacterial DNA load during the first year of treatment [124].

The antiseptic chlorhexidine has been also evaluated for preparation of periodontal implantable anti-microbial delivery devices. Yue *et al.* [92] incorporated chlorhexidine in PLGA microparticles prepared by the single emulsion-solvent evaporation technique. To increase the encapsulation efficiency and to optimize the release characteristics and bioactivity of the anti-microbial agent, chlorhexidine was incorporated as a free base, as digluconate salt and as an inclusion complex with methylated-β-cyclodextrin (MβCD) or hydroxypropyl-β-cyclodextrin (HPβCD). The chlorhexidine encapsulation efficiency was clearly improved by the use of cyclodextrins, and better results were obtained with microparticles prepared with the more hydrophilic HPβCD when free base drug was used and with more lipophilic MβCD when drug digluconate salt was included. Furthermore, the results indicated that the use of cyclodextrins prevented premature drug release, avoiding the initial burst effect, and favored the controlled drug release.

Other antibiotics were also evaluated for microencapsulation and treatment of periodontal infections. In 2007, Ferraz *et al.* [106] developed microspheres composed of alginate/hydroxyapatite and loaded with amoxicillin, erythromycin, and amoxicillin-clavulanic acid to produce injectable drug delivery microspheres for treatment of periodontitis and simultaneously, to initiate the osteointegration process. Microspheres were prepared by ionic gelation, by use of a suspension of hydroxyapatite nanoparticles in sodium alginate with a high α-*L*-glucuronic acid content, with CaCl_2_ as a cross-linking agent. Release studies performed over 28 days showed that hydroxyapatite pellets prepared by compaction and subsequent milling, released all of the antibiotic during the first hours. Microspheres prepared with two types of hydroxyapatite have been found to have an adequate sustained drug release profile. Furthermore, osteoblasts proliferated well in both types of microspheres, and cell growth was enhanced in the presence of antibiotics. Microspheres loaded with erythromycin had the most beneficial effect.

Emulsion-solvent evaporation/extraction methods have been widely used for microsphere preparation. For example, in a recent study, Srirangarajan *et al.* [126] used a water-in-oil/oil-in-water (W/O/W) double-emulsion solvent evaporation technique to prepare doxycycline loaded PLGA/PCL– blend microspheres, and compared their efficacy with a commercially available gel. Doxycycline PLGA/PCL–blend microspheres showed an initial burst effect that resulted in a higher initial drug release than from commercial gel. However, after the first hours of release, microspheres provided a more sustained release. *In vivo* clinical studies confirmed that patients treated with microspheres as coadjuvant treatment to SRP showed improvements in all clinical and microbiological parameters.

The clinical evaluation of local delivery devices in periodontal therapy is a controversial topic. In 2006, Greenstein [127] published a critical review of the clinical improvements obtained by the use of controlled drug release devices used for local delivery of drugs in periodontal diseases as monotherapy or in combination with the conventional scaling and root planning (SRP). The author suggested that results of such studies need to be reinterpreted with respect to their statistical and clinical significance, because these terms may not be necessarily interchangeable. Thus, statistically significant responses to therapy recorded in clinical trials may not be clinically significant. It has also been reported that the use of minocycline microspheres in monotherapy achieved results that were not statistically different from results obtained with SRP. Furthermore, in many patients who responded to conventional SRP treatment, the routine application of adjunctive chemotherapy during active therapy may not be advantageous. In contrast, numerous researchers have successfully used local delivery in patients who did not respond to SRP alone. As a result, Greenstein proposed that the clinician's decision to use local drug delivery as an adjunct to SRP must be determined on an individual case basis, and factors that should be considered include data reported in the literature, clinical findings, desired clinical outcomes, and the patient's medical and dental history. The variety of delivery systems and polymers, dosage regimes, instrumentation used during mechanical debridement, patient populations, stage of the disease or location of the infection and other multiple factors such as measurement of disease regression, have been analyzed to demonstrate the outcome of these therapies. Future research should follow generally recognized and standardized large-scale methods to obtain an objective evaluation of human clinical trials in where microparticles are used.

## 3. Microparticulate Drug Delivery Systems and Dental Route of Administration

The dental route of administration consists of the intracanal delivery of drugs, usually antibiotics, to eliminate the bacterial infection, and nonsteroidal anti-inflammatory drugs (NSAIDs), to control the post-treatment pain [46,52,128-130]. The medication is placed into the canal before the permanent cap is installed. Within this context, microparticles could be inserted into the root cavity (enlarged and shaped by dental instruments to a depth of 4–5 mm) with a syringe, to prevent bacterial growth.

The residence time for a drug in the oral cavity is usually quite short, and the bioavailability of most drugs in the mouth is therefore low. However, dental administration implies a small, relatively closed system. Inside the canal, the drug delivery system remains limited to the cavity and should be non-toxic, provide sustained drug release, be compatible with dentine, should have low surface tension, good canal fit, and be easily removable. The histocompatibility of materials for endodontic therapy is also important since they are in contact with internal tissues through the root apex of the tooth.

Another advantage of dental administration of drugs is that the canal root is partly surrounded by tissue that is poorly irrigated and with low enzymatic activity. Most of the delivery surface is the teeth themselves. On the other hand, canal dressing does not require prolonged administration or chronic use of drugs. Furthermore, dental delivery also allows removal the delivery system once the therapy has terminated, which prevents tissue sensitization and other allergic reactions. The root canal cavity is therefore an attractive route of delivery, especially in the case of endodontic failure.

Many systems of different shapes (sharp cones, long pins or screws) have been proposed for placement in the root canal as a temporary dressing for local drug delivery and healing. Antibiotic-loaded polymer matrices have been developed by several researchers for treatment of endodontic disease. Sustained release devices such as monolithic fibers made from ethyl vinyl acetate [131], and needle-shape systems [132] that provide different rates of release have been prepared. Huang *et al.* [132] conducted an interesting study in which chlorhexidine-loaded devices were prepared with ethyl cellulose, to form a needle-like device suitable for insertion in the root canal. A theoretical model for *in vivo* drug release, based on a diffusion model of drug release, has been proposed considering parameters such as morphology, drug absorption and binding, the limited volume of release medium, and temporary sealing of root canals.

Considerable efforts are being undertaken in the development of novel sustained release systems for root canal treatment. As a result, many prototypes made from biodegradable polymers have been proposed. Chitosan, PLGA and polymethylmethacrylate have been used as coatings for absorbent paper to examine the controlled release of chlorhexidine digluconate loaded-paper points [133]. Biocompatible amoxicillin-collagen sponges have also been developed by Luzardo *et al.* [134], who concluded the positive effect of cross-linking on drug release, water uptake, mechanical properties and cell compatibility of sponges made of collagen and Gantrez^®^ loaded with amoxicilline, in comparison with non-crosslinked sponges.

Other interesting alternatives have been investigated. Pagonis *et al.* [135] used a combination of light and poly(lactic-co-glycolic acid) (PLGA) nanoparticles (150–200 nm) loaded with the photoactive drug methylene blue to treat infections caused by *E. faecalis*. The photodynamic therapy was applied *in vitro* with extracted human teeth infected with *E. faecalis*. Nanoparticles prepared by the solvent displacement technique were placed in the infected root canal. Photodynamic treatment of root canal bacteria showed that survival relative to control levels of CFU (colony forming units) was 15.2% for light activation of drug-loaded particles, however, no data on the encapsulation efficiency or drug release were provided. In another study, conducted by Kishen *et al.* [136], non-loaded chitosan nanoparticles were prepared by ionic gelation of sodium tripolyphosphate of size ranging between 60–120 μm and positive surface charge of 40 mV. Chitosan nanoparticles were found to display a high degree of antibacterial activity against *E faecalis* in *in vitro* culture, and good compatibility with the endodontic sealer. The authors hypothesized that the maximum reduction in bacterial adherence to the dentin was achieved by the electrostatic interaction between the negatively charged dentin and cationic nanoparticles, which substantially inhibited the adherence of bacterial cells to dentin substrate, thus preventing biofilm formation. Although the adhesion may be weak, nanoparticles may also interact directly with microbes when they re-enter the root canal. Chitosan and some derivatives are known to inhibit growth of microbes by different mechanisms [137-139].

Shrestha and other colleagues [140] used similar nanoparticles prepared with chitosan for use in combination with ultrasound to remove the bacterial biofilm and subsequently, to inhibit bacterial adherence by the formation of cavitation bubbles, designed to push the nanoparticles into areas inaccessible to conventional treatment. Dentin sections of 1.5–3 mm were exposed to nanoparticle suspensions and application of high-intensity ultrasound. The efficacy of ultrasonic irrigation for penetration of nanoparticles into the dentinal tubules was demonstrated and the particles were found to enter as far as 100 μm, although their internal distribution was not homogeneous. Chitosan has also grown in importance in recent years for the development of new types of cement for root canal filling or pulp-capping, alone or in combination with other materials [141,142].

Recent research has unveiled some novel formulations based on microparticles, which appear promising for accessing the conventionally inaccessible parts of the root canal. Sousa *et al.* [143] developed amoxicillin-loaded microparticles based on mixtures of PLGA/zein. Microparticles (5–38 μm) prepared by spray-drying have shown different drug release profiles, depending on the composition of the formulations. Drug characteristics also played an important role in the release. The antimicrobial activity of amoxicillin was preserved when it was encapsulated, and *in vitro* antimicrobial activity studies demonstrated that the MIC_90_ against *E faecalis* was achieved over 6 days. The spongy conformation of particles on contact with water facilitated the complete removal of the formulation from simulated root canals. In contrast to the huge amount of periodontal literature on drug loaded-microparticles, to our knowledge this was the first study that considered that microparticles may exhibit appropriate features for use as temporary fillings for root canal disinfection.

However, the limitations of these studies, performed only under *in vitro* conditions, must be recognized.

## 4. Periodontal and Dental Tissue Regeneration

Other biomedical applications may benefit in the near future from the advantages of microencapsulation. Whereas the previously described microencapsulated formulations may serve to fulfil the function of hindering the growth and invasion of bacteria in the empty chamber, regeneration of teeth and restoration of the physiological function of lost tissues are also important concerns in endodontics.

Dental tissue engineering is aimed at regenerating complex structures such as bone, teeth and soft dental tissues (pulp). As a consequence of a local infection, such as periodontal disease, or as a result of dental deterioration, bone loss in the oral cavity is associated with an alteration in the balance between bone formation and destruction.

Repair of defects in the periodontal ligament (the fibrous connective tissue) and surrounding bone, and the regeneration of a whole tooth by delivery of growth factors, gene therapy or cellular implantation present typical difficulties associated with the bacteria that occur naturally in the oral cavity and the complexity of teeth, which are composed of multi-layered matrices (enamel, dentin and cementum layers) and soft tissue (the dental pulp) [144-147]. In addition, the pulp tissue is richly innervated and blood and lymph vessels are present. The homeostasis in the pulp depends on the close relationship between the vascular and the nervous system. In many cases, current treatments for periodontal bone defects do not restore normal functions to tooth-supporting structures [148,149]. Regeneration of such functional and well organized structures is a difficult task. Nonetheless, successful results as regards the regeneration of dental pulp have been obtained *in vitro* and *in vivo* with human dental pulp stem cells seeded onto scaffold matrices made of collagen, PLGA or gelatin [150-153]. The capacity of dental pulp stem cells to differentiate into osteoblastic, fibroblastic and cementoblastic cells when cultured in the appropriate environment determines their great potential in the regeneration of periodontal systems [154-157]. The real interest in biocompatible microparticles in the management of periodontal or dental regeneration is derived from their ability to serve as controlled drug delivery systems that can release growth factors to promote tissue regeneration [158-161]. Localized delivery of growth factors from microparticles may also protect them from the proteolytic environment at the desired site of action, thus facilitating their adsorption and preventing their rapid diffusion from the tissue, as generally occurs when solutions of growth factors are injected directly into the site. Growth factors generally have short half-lives *in vivo* and low oral bioavailability. Sustained release formulations can provide means of controlling polypeptide levels at the site of action and are also effective, safe and well-accepted by patients. Thus, individualized microparticulate formulations for controlled release of growth factors could be developed depending on each clinical situation or release kinetics requirements [159,162-164]. Properties such as biodegradability, size, surface area, porosity, hydrophobicity and release pattern can be optimized by modifying the composition of polymers (nature or molecular weight), or the methods of preparation. Periodontal tissue regeneration is one of the fields of biomedicine that can obviously benefit from the specific properties and versatility of microparticulate delivery systems.

**Figure 4.**
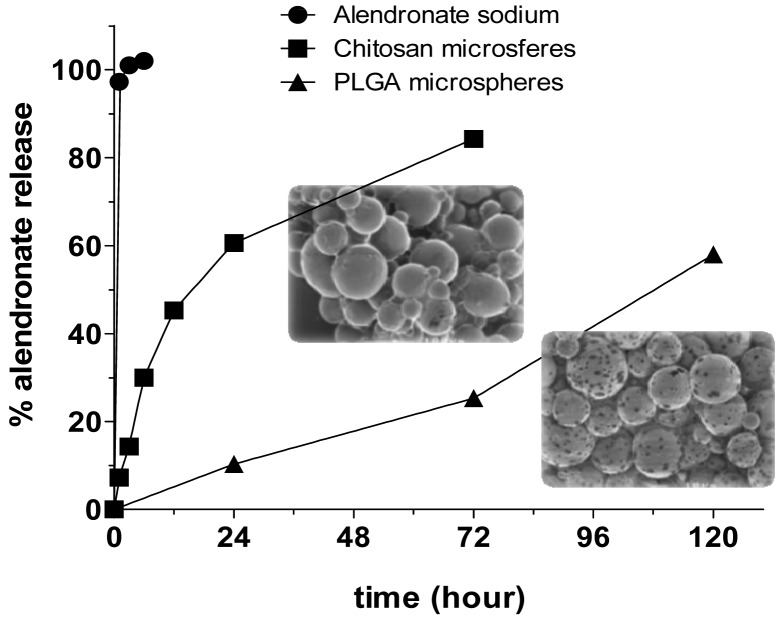
Alendronate release profiles obtained from alendronate sodium powder and alendronate loaded chitosan and PLGA microspheres. Modified with permission from Informa Healthcare [96].

Restoring the damaged pulp in dentin structures has traditionally been carried out with calcium-based biomaterials. However, the presence of a small population of progenitor cells in the dental pulp and periodontal ligament of teeth able to differentiate depending on the effect of appropriate signals has led to the development of novel methods to regenerate the tissues of the endodontium, including the use of scaffold biomaterials. Suitable microparticulate carriers for bioactive molecules such as growth factors have also been used for purposes of regeneration. Polypeptides found to have an important effect in regeneration of pulp dentin include TGF-β (transforming growth factor beta family), BMP-2, -4, -7 (bone morphogenetic proteins), FGF-2 (fibroblast growth factor-2), and DMP1 (dentin matrix protein 1). New strategies, based on the release of morphogenetic signals from microparticles to induce specialization of the stem cells to repair the new tooth tissue, are therefore emerging [165,166]. Dentin formation was observed after 12 weeks when TGF-β1-loaded PLGA microparticles and calcium phosphate cement were placed in the root canal of goats [165]. In this study, protein was physically adsorbed onto PLGA microparticles prepared by emulsion-solvent-evaporation. Histological analysis revealed evident formation of dentin in comparison with negative controls. The odontogenic effect of TGF-β1 was concentration-dependent although PLGA particles and calcium phosphate cement showed similar levels of regenerated dentin as the samples with lower levels of TGF-β1. Although the PLGA copolymer is one of the most extensively used for dental tissue engineering, gelatin, collagen and other polymers have also been used to regenerate the periodontal ligament surrounding teeth [167-171]. For example, implantation of chitosan and poly(lactide-1coglycolide) acid (PLGA) microspheres loaded with alendronate sulphate (AS) has been used by Samdancioglu *et al.* to treat osteolysis [96]. AS-loaded PLGA and chitosan microspheres were prepared by solvent evaporation and emulsion polymerization methods, respectively. All microspheres were spherical in shape but the PLGA microspheres were smaller (39–74 μm) than chitosan systems (110 μm) and differed as regards their surface properties, loading efficiency and release. Release of AS was faster from chitosan microspheres (85% within 75 h), with first order release kinetics, while PLGA microspheres release only 25% of AS within 75 h, giving zero order release kinetics (Figure 4). According to results obtained in *in vitro* studies, the authors concluded that AS-containing microspheres appear to be promising for local application in osteolysis.

Development in the field of dental tissue engineering has mainly focused on the area of periodontics (periodontal ligament, root cementum and lost alveolar bone) and oral and maxillofacial bone defects [172], resulting from periodontitis. In this context, various drug delivery devices made from different materials (PLGA, collagen, chitosan, silk, fibrin, poly-ε-caprolactone, ceramics or calcium phosphate cements have been tested by use of different pharmaceutical formulations such as membranes, hydrogels and foams [168,173-181]. However, approaches for the repair of dental tissues with microparticles are unfortunately scarce [168,182]. It is difficult to produce ideal treatments in regenerative endodontics because of the complexity of the dental systems.

Some synthetic flavonoid drugs such as ipriflavone have been used in the treatment of diseases involving bone loss, e.g., osteoporosis [183,184] because they favor osteoblast activity and inhibit bone reabsorption [185]. Perugini *et al.* [67] prepared PLGA microspheres containing ipriflavone by emulsion-vaporation. The effect of technical preparation variables on the morphology, size and drug loading capacity and *in vitro* and *in vivo* evaluation of the ability of ipriflavone charged microspheres for local treatment of bone mandibular loss were studied. The authors used an animal model of mandibular osteoporosis and reported that microspheres showed good *in vivo* tolerance when administered in the alveoli after extraction of the tooth. PLGA has proven to be a good bioactive material in endodontic practice when used with α-tricalcium phosphate, which is commercially used in dentistry as canal filling. Matrices, composed of polymer and bio-ceramin, have been found to improve the mechanical properties of PLGA and bone formation [186,187].

Chen [167] used an interesting approach to repair the tissues of the periodontium. Two growth factors (BMP-2 and IGF-1) were co-encapsulated in glycidyl methacrylated dextran-cross-linked gelatine. This system was integrated in scaffolds made of the same material to avoid interactions between the two proteins. Release studies demonstrated that protein release was significantly slower when the growth factors were loaded into microspheres, but the release patterns were similar to those in the scaffolds containing either BMP-2 or IGF-1 alone. Incorporation of the growth factors enhanced the induction of osteoblastic differentiation in *in vitro* culture of periodontal ligament fibroblasts. In another study to induce the regeneration of periodontal tissues in beagle dogs, a collagen sponge containing basic fibroblast growth factor (bFGF) loaded in gelatine microspheres were combined in a “sandwich membrane” configurated and implanted in bone defects associated with periodontal injury. After 4 weeks, the collagen fibers in bFGF- treated dogs showed clear signs of regeneration, which led to the functional recovery of periodontal ligament. Vascularization and osteogenesis were observed by histological analysis, whereas the non-treated group exhibited epithelial downgrowth and root resorption to enhance regeneration of the connective tissue [167].

Osteoinduction was observed after treatment with microspheres loaded with succinylsulphatiazole or naproxen [99]. Veronese and colleagues prepared microspheres with two polyphosphazenes bearing amino acid side groups, for bone regeneration. Polyphospazenes have osteoinductive properties similar to other materials such as hydroxyapatite and bovine bone. Two types of biodegradable polyphosphazenes substituted with alanine ethyl ester or phenylalanine ethyl ester were synthesized and used to fabricate biodegradable microspheres. The *in vitro* and *in vivo* results showed the usefulness of polyphospazene microspheres for use in bone regeneration and drug control release. The use of 50 μm microspheres mixed with hydroxyapatite (ratio 10/90) facilitates bone regeneration and enables prolonged drug control delivery to be obtained.

Surgical implantation of membranes containing microparticulate carriers for controlled release of growth factors and induction of bone formation have been used successfully by Chenin [170]. Biodegradable dextran-co-gelatine hydrogel microspheres loaded with recombinant human bone morphogenetic protein-2 (rhBMP2) were prepared with the aim of maintaining the protein biological activity and achieving long-term control release. The microspheres (20–40 μm) maintained the *in vitro* rhBMP2 release for more than 10 days. Cytological studies showed that rhBMP2 loaded microspheres were better at promoting the proliferation and osteoblastic differentiation of periodontal ligament cells than an aqueous solution of protein. *In vivo* studies indicated that scaffolds or functionalized membranes that incorporate rhBMP2 in dextran-co-gelatine hydrogel microspheres induced greater regeneration of periodontal tissue than scaffolds or general membranes soaked in a solution of rhBMP2.

Other delivery strategies are based on hydrophobic microspheres coated with collagen to improve cell attachment and spreading. Aishwarya *et al.* [188] proposed the use of PCL-microparticles coated with acetylated collagen as scaffolds in periodontal bone regeneration and for in situ release of doxycycline hydrochloride. Doxycycline-loaded PCL microparticles were prepared by emulsion-evaporation, and coated with acetylated collagen containing doxycycline to increase the drug availability. *In vitro* drug release studies showed that drug release lasted for more than 10 days, and *in vitro* fibroblast culture studies revealed that coated microparticles may be a good substrate for cell proliferation and regeneration.

Finally, chitosan has been used alone or in combination with inorganic components of human hard tissues, such as tricalcium phosphate [189], hydroxyapatite [105] and calcium sulfate [190,191] to improve bone growth. For example, Chang [190] *et al.* proposed the use of chitosan microsphere composites containing CaSO_4_ and platelet-rich plasma (PRP). Microspheres were fabricated by means of a high voltage electrostatic field system, using mixtures of chitosan/CaSO_4_ or chitosan/CaSO4/PRP. Three different formulations were prepared: chitosan/CaSO_4_ microspheres and chitosan/CaSO_4_ or chitosan/CaSO_4_/PRP microspheres mixed with thrombin. *In vivo* studies developed in a pig model showed that microspheres mixed with thrombin produced greater increases in new bone formation along with fibrous tissue regeneration in comparison with chitosan/CaSO_4_ microspheres without thrombin. The chitosan/ CaSO4 microspheres with thrombin produced the most abundant fibrous collagen matrices around the defects. The authors concluded that chitosan/CaSO_4_-based microspheres with thrombin could be used successfully in regenerating new bone around the alveolus bone area.

Despite the evident efforts in dealing with various difficulties associated with regenerating oral and dental tissues by use of microparticles, the field of regenerative periodontology, particularly regenerative endodontics, is in its infancy. In the future, the clinical efficacy and the potential routine use of growth factors releasing microspheres for dental and oral tissue engineering will depend on how closely multidisciplinary research teams can contribute their own areas of expertise to expand this field of research.

## 5. Efficacy and Safety

The efficacy of periodontal therapy and root canal treatment or re-treatment can be considerably enhanced by the use of appropriate vehicles such as microparticles. Although no set criteria are available for the design of an appropriate microparticulate carrier system for oral and dental applications, it is reasonable to assume the following general guidelines: adequate concentrations of antibiotics, anti-inflammatory drugs or growth factors must be appropriately released inside the periodontal pocket or the root canal cavity. In addition, microparticle formulations should provide several advantages: 1). The materials should be non-toxic and well standardized. The biocompatibility of some of the natural or synthetic polymers extensively used for microparticles preparation have been demonstrated elsewhere [5,192-196]; 2) Formulations should release the active substance in a precise sustained manner; 3) They should not necessarily be resorbable, but should be easy to implant in and remove from oral or dental cavities; 4) In addition, their small size (microns) will allow their insertion in the root canal cavity (depth of 4–5 mm) or in the periodontal pocket; 5) Evidently, the microparticulate formulations should be themselves free from transmittable disease or viral contaminants without eliciting an over-inflammatory response to the polymers in the host, and 6) Other efficacy issues associated with the use of microparticulate systems for dental applications include the retention of the formulation at the site of action, mainly the periodontal pocket. The use of bioadhesive microparticles may enhance healing by increasing the residence time of formulation. The self-retentive properties of microspheres and polymers have been exploited for oral treatments to enhance drug bioavailability and to prolong the absorption time [15,197].

Safety is an important concern with any new form of drug release system. The safety and biocompatibility of microparticulate carriers for dental application has not yet been extensively studied, and very few studies have addressed these issues [91,117,118,120]. Safety assessments include adverse events such as dental infection, headache, tooth sensitivity, dental pain, gingivitis, increased periodontitis, stomatitis, changes in vital signs (blood pressure, temperature or heart rate) or complications associated with over-immune response. In general, the results of human trials have indicated that microparticulate dosage forms are safe and well tolerated. In addition, much is known about the safety, biodegradability and biocompatibility of polymers, such as PLGA, used for microsphere preparation. The safe management of microparticles for periodontal and dental application involves a complete understanding of this particular scenario and the cellular and molecular mechanisms involved in periondontal and dental healing. It is also essential to comprehend the interaction between microparticles and the biological system. It is generally assumed that characteristics such as size, surface charge, chemical composition, stability and biodegradation, porosity, hydrophobicity, roughness and ligands that may attach to the surface are of particular importance as regards the interaction between microparticles and cells [198-200]. Furthermore, the spherical shape, low size and the non-rigid conformation of microparticulate carriers may be an advantage once formulations are implanted in dental cavities. Since endodontic and periodontic procedures mainly attempt to preserve already damaged and inflamed tissue, localized adverse effects resulting from friction or abrasion could impair the healing of periodontal lesions. As regards the physiological features and pharmacokinetics of local delivery though periodontal pockets and empty canal cavities, microparticulate carriers may be considered as relatively safe for localized drug delivery in periodontal and dental applications. Finally, the versatility and safety of microparticulate carriers demonstrated in other biomedical fields for release of substances confirm their potential usefulness for local delivery in oral and dental applications.

## 6. Conclusions

The management of periodontal diseases and endodontic failure has traditionally focused on the use of anti-infective irrigating solutions and the use of mechanical procedures to eliminate infectious tissue and to hinder disease progression. The use of adjunctive microparticle-based controlled drug delivery systems to achieve and maintain effective concentrations of therapeutic agents within the site of action, namely the periodontal pocket, dental cavity, or surrounding bone, have been found to increase the effect of conventional mechanical therapies, with clear clinical benefits.

The controlled release from polymer microparticles enables therapeutic levels of drugs to be reached in periodontal or dental treatments by modifying the physicochemical properties of microspheres or the appropriate selection of materials for the formulation. The appropriateness of microspheres will depend on such physicochemical features, and therefore on release kinetics, site of infection and pathogens, drug of choice, and in the case of tissue regeneration, the ability of the formulation to induce formation of the desired tissue.

In light of the studies discussed in this review, drug-loaded microspheres can be considered as good carriers for the controlled release of several active substances, antibiotics or growth factors suited to such specific sites as periodontal pockets or dental cavities.

Site specific administration of simple injectable formulations of microparticles in the periodontal pocket or in the root canal cavity would reduce the number of treatment sessions for patients, and may serve as an adjuvant to surgical protocols offering a means of saving teeth, the ultimate goal.

The success of drug or growth factors-loaded microparticles as adjunctive therapies remains to be determined as more clinical data become available and reviewed. Recent approaches, particularly in the field of periodontal diseases and periodontal tissue engineering show promising results with respect to the introduction of microparticles as complementary treatment. Microencapsulation technologies may open up new directions in the field of periodontal and dental therapy.

## Figures and Tables

**Table 1.** Overview of most widely studied polymers for preparation of microspheres for periodontal applications and studies where they were used.

**Polymer**	**Drug**	**Comments**	**Ref.**
Polyphosphazenes	Naproxen Succinylsulphatiazole	Extraction/evaporation of solvent (Size 30-80 μm) Drug content 5%; 800 h release. Satisfactory therapeutic levels; *In vivo* study in rats	[99]
*L*-PLA *D*,*L*-PLA *DL*-PLG	Tetracycline	Extraction/evaporation of solvent (O/W, O/O and W/O/W) Encapsulation yield <3% Controlled release profiles for 12 days.	[82]
Gelatin	Propolis	Spray-drying Microparticles in syringable/injectable semisolid Formulation Period of release: over 7 days.	[107]
Alginate/chitosan	Minocycline	Microcapsules (Ionic gelification) Determination of minocycline in gingival crevicular fluid in patients. 7-days release.	[101]
Polyhydroxybutirat e-co-hydroxyvalerate (PHBV)	Tetracycline	Microcapsules (W/O/W emulsion). Particle size 322-538 μm. Study the influence of surfactants (PVA, gelatine) and drug form on encapsulation efficiency andrelease kinetics	[108]
PLGA	Minocycline	Clinical study using microspheres (20–60 μm) in patients with chronic periodontitis. Evaluation of efficacy and safety	[91,125]
PLGA	Minocycline	ICTP crosslinked with pyridinoline. Microspheres used in periodontal treatment complementary to scaling and root planning. Clinical evaluation. Biomarkers evaluation of bone resorption	[116]
PLGA/PCL	Doxycicline	Microspheres prepared by double emulsion (W/O/W) Physic-chemistry characterization *In Vitro* and clinical evaluation. Chronic periodontitis	[93]
PLGA	Minocycline	(Arestin ™ /Orapharma Inc.). Adjuvant to mechanical treatment of peri-implantitis lesions; Clinical evaluation; Comparison with clorhexidine gel.	[117,118]
PLGA/PCL	Doxycycline	Microspheres; solvent/evaporation technique(W/O/W). Comparison with chlorhexidine gel. Study of characterization (size, release, antimicrobial activity) and microbiologic and clinical parameters.	[126]
PLGA	Minocycline	Arestin TM; Oralpharma Inc. Use of microspheres in adjuvant surgical therapy in chronic and severe periodontitis	[109]
PLGA	Minocycline	Arestin ™ /Orapharma Inc. Microspheres as adjuvant in SRP treatment in smoking people with chronic periodontitis	[123]
Chitosan	Tetracycline	Beads prepared by ionotropic gelation. Influence of formulation variables in microspheres parameters (drug content, yield, particle size and morphology.	[102]
Chitosan CMCchitosan	-	W/O emulsion Microspheres (50 μm) dispersed in a PVA film Biocompatibility in rats	[110]

## Abbreviations

PD: periodontal disease
EF: Endodontic failure
SRP: scaling and root planning
GCF: gingival crevicular fluid
PLA: poly(lactide)
PLGA: poly(lactide-co-glycolide)
PCL: poly(ε-caprolactone)
CMC: carboxymethyl cellulose
PHBV: polhydroxybutirate-co-hydroxyvalerate
PEO-PPO-PEO: polyethylene oxide/polyprolylene
ICTP: pyridinoline cross-linked carboxy-terminal telopeptide of type I collagen
MβCD: methyl β-cyclodextrin
HPβCD: hydroxypropyl β-cyclodextrin
rhBMP2: recombinant human bone morphogenetic protein-2
PRP: platelet-rich plasma
TGF-β: transforming growth factor β family
IGF-1: insulin-like growth factor-1
bFGF: basic fibroblastic growth factor
DMP1: dentin matrix protein 1
